# Acute tubulo-interstitial nephritis leading to acute renal failure following multiple hornet stings

**DOI:** 10.1186/1471-2369-7-18

**Published:** 2006-11-21

**Authors:** Aman Sharma, Ajay Wanchu, V Mahesha, V Sakhuja, Pradeep Bambery, Surjit Singh

**Affiliations:** 1 Department of Internal medicine, Post Graduate Institute of Medical Education and Research, Chandigarh, India; 2 Department of Pathology, Post Graduate Institute of Medical Education and Research, Chandigarh, India; 3 Department of Nephrology, Post Graduate Institute of Medical Education and Research, Chandigarh, India

## Abstract

**Background:**

Hornet stings are generally associated with local and occasionally anaphylactic reactions. Rarely systemic complications like acute renal failure can occur following multiple stings. Renal failure is usually due to development of acute tubular necrosis as a result of intravascular haemolysis, rhabdomyolysis or shock. Rarely it can be following development of acute tubulo-interstitial nephritis.

**Case presentation:**

We describe a young male, who was stung on face, head, shoulders and upper limbs by multiple hornets (Vespa orientalis). He developed acute renal failure as a result of acute tubulo-interstitial nephritis and responded to steroids.

**Conclusion:**

Rare causes of acute renal failure like tubulo-interstitial nephritis should be considered in a patient with persistent oliguria and azotemia following multiple hornet stings. Renal biopsy should be undertaken early, as institution of steroid therapy may help in recovery of renal function

## Background

Insect stings and bites are known to cause a variety of allergic reactions and direct toxic effects. Stinging insects are classified as hymenoptera which includes apids (honey bees, Africanized bees) and vespids (wasps, yellow jackets & hornets). Hornet stings are generally followed by minor allergic reactions and rarely anaphylaxis [1,2]. However multiple stings can sometimes lead to angioedema, vasculitis, encephalitis and acute renal failure [3]. Usually acute renal failure is due to acute tubular necrosis secondary to intravascular hemolysis, rhabdomyolysis or shock [4-7]. Rarely it can be due to the development of tubulo-interstitial nephritis [8]. In this report we describe a young male who had multiple hornet stings (Vespa orientalis) and developed acute renal failure as a result of tubulo-interstitial nephritis.

## Case presentation

An 18 year old male was stung on face, head, shoulders and upper limbs by multiple hornets while sitting under a tree. He developed severe pain at the site of the bites and was managed at local hospital. A day later he developed oliguria and by next day became anuric and developed nausea and vomiting, following which he was referred to our hospital. At admission, on examination, he was conscious and had mild pallor. The systemic examination was normal. Twenty-seven sting marks were found on face, shoulders and upper limbs.

Investigations revealed hemoglobin of 7.6 g/dl platelet count of 529,000/mm^3^; TLC of 16200/mm^3 ^with peripheral blood film revealing microcytes, micro-ovalocytes, macrocytes with hypochromia. Blood biochemistry revealed urea 310 mg/dl, S. creatinine of 9.2 mg/dl. Serum CPK and transaminases (AST & ALT) were within normal limits. He had normal urinalysis. There was no evidence of intravascular hemolysis as urine was negative for hemoglobin and plasma hemoglobin was less than 20 mg/dl. Ultrasound of abdomen revealed normal sized kidneys.

**Figure 1:**
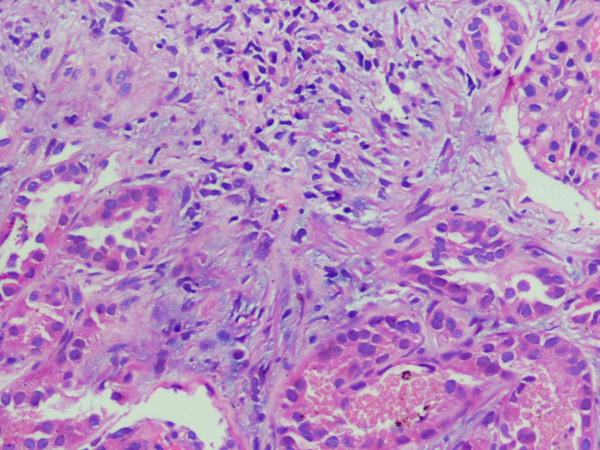
Histopathology of kidney core biopsy. 10× HE stain sections showing interstitial infiltrate comprising lymphomononuclear cells along with eosinophils suggestive of tubulo-interstitial nephritis.

His renal failure was managed by hemodialysis. However despite repeated hemodialysis, his urea and creatinine remained persistently high so kidney biopsy was undertaken after two weeks. It revealed 12 normal glomeruli, focal interstitial infiltration with eosinophils and lymphocytes with hyaline and pigmented granular casts in the tubules (fig 1). These findings were consistent with tubulo-interstitial nephritis. In a focal area, the interstitial inflammationwas accompaniedbyinterstitial edema and few dilated tubules lined by flattened epithelium. This may besuggestive ofaccompanyingfocal/patchy acute tubular necrosis.

He was started on prednisolone 50 mg/day. Gradually his urine output increased to more than 1.5 liter/day and he was discharged when his serum creatinine decreased to 3 mg/dl. His nutritional anemia recovered on follow up with supplementation of Iron, Vitamin B12 and folic acid. His steroids were tapered over next four weeks and he recovered completely.

## Conclusion

Stinging insects are members of the Order Hymenoptera of class Insecta. In most instances, insect stings are only followed by allergic reactions but sometimes intravascular hemolysis, rhabdomyolysis, thrombocytopenia [9], acute tubular necrosis, acute hepatic injury[10,11], and myocardial infarction [12] have been reported. Their venom is a concentrated mixtures of complicated active components, such as melittin, apamine, phospholipases, hyaluronidase, acid phosphatase, histamine, and kinin. These have direct and indirect hemolytic effects, neurotoxic and vasoactive properties, which can cause intravascular hemolysis and rhabdomyolysis [4]

There have been various case reports of acute renal failure in patients with Hymenoptera stings. Initially it was attributed only to tubular necrosis(ATN) either due to shock or pigment nephropathy due to intravascular hemolysis or rhabdomyolysis [5-7,13]. Three of the five patients with hornet bite (V. orientalis) and renal failure reported by Sakhuja et al had histopathological evidence of ATN[5].

Mejia et al also reported five cases of acute renal failure following African bee stings. Post mortem renal biopsy done in one of these patients showed dense proteinaceous casts of collecting tubules and ascending loop of henle (probably due to shock), arterial nephrosclerosis (due to underlying hypertension) and unexplained membranous glomerulonephritis. Rhabdomyolysis and ischemia was thought to be the most probable cause of renal lesions [13]. There were reports of renal failure without any evidence of hemolysis and shock earlier but as renal biopsy was not done, the cause could not be ascertained and it was postulated to be due to the direct toxic effect of the venom [14]. Sakhuja et al had also postulated that direct toxic effect can not be directly excluded. Vikrant et al reported three case of acute renal failure following wasp bite but only two had evidence of intravascular hemolysis [15]. So obviously there are causes other than ischemic/toxic acute tubular necrosis which are responsible for development of acute renal failure in such patients.

Zhang et al reported for the first time that acute tubulointerstitial nephritis could lead to acute renal failure in these patients[8]. It was postulated by them that, as these patients have other allergic manifestations like serum sickness-like reaction, delayed skin eruption, and possible vasculitis and as many components of the insect venom are polypeptides so theoretically, these allergens can mediate various types of renal injuries, such as immune complex-mediated glomerulonephritis, interstitial nephritis, renal microangiitis, or vasculitis.

Subsequently Chao et al reported a case with both acute interstitial nephritis and tubulopathy after wasp bites [16]. The role of steroids in the setting of tubulointerstitial nephritis has also been discussed in these two reports[8,16]. Our patient also had acute renal failure due to acute tubulointerstitial nephritis which responded to steroid therapy. The cause of acute tubulointerstitial nephritis is not clear. There was no history of any nephrotoxic drug intake so it could be due to hypersensitivity to the venom. This patient also had anemia at presentation but there was no evidence of hemolysis and the peripheral blood film was consistent with nutritional anemia which recovered on follow up with appropriate supplementation.

In conclusion, rare causes of acute renal failure following hornet bite like tubulo-interstitial nephritis should be considered in a patient with persistent oliguria and azotemia and renal biopsy should be undertaken as institution of steroid therapy may help in recovery of renal function and preventing development of interstitial fibrosis.

## Competing interests

The author(s) declare that they have no competing interests.

## Authors' contributions

AS, AW, PB and SS managed the patient, conceived the idea and drafted the manuscript. VS got the renal biopsy done. VM did the histopathological examination of the renal biopsy. All the authors read and approved the final manuscript.

## Pre-publication history

The pre-publication history for this paper can be accessed here:


